# Editorial: Natural Products and Hepatic Health: Light and Shadows

**DOI:** 10.3389/fphar.2022.868207

**Published:** 2022-02-25

**Authors:** Silvia Di Giacomo, Oscar Briz, Annabella Vitalone, Antonella Di Sotto

**Affiliations:** ^1^ Deptartment of Physiology and Pharmacology “V. Erspamer”, Sapienza University of Rome, Rome, Italy; ^2^ Laboratory of Experimental Hepatology and Drug Targeting (HEVEFARM), IBSAL, University of Salamanca, Salamanca, Spain; ^3^ Center for the Study of Liver and Gastrointestinal Diseases (CIBERehd), Carlos III National Institute of Health, Madrid, Spain

**Keywords:** herbal extracts, hepatoprotection, hepatotoxicity, liver disease, gut microbiota, waste, polyphenols, antioxidant

The liver is an essential organ for survival, being involved in the regulation of several physiological functions, including synthesis and storage of nutrients, detoxication, and decomposition of xenobiotics and toxicants, along innate immunity (Marin et al., 2020). Hepatic ailments often compromise global wellness, leading to severe reactions and troubles, and sometimes to death (Zhang et al., 2018). Indeed, with approximately 2 million deaths per year worldwide, liver diseases represent one of the major causes of death globally and a major threat to public health; among them, viral hepatitis is the most common while nonalcoholic fatty liver disease (NAFLD) is the most rapidly growing contributor to mortality and morbidity. The NAFLD increased incidence seems also to contribute to the risk of cirrhosis and liver cancer (Asrani et al., 2019).

In the past decades, several efforts have been made to fight liver diseases; however, limitations in finding more effective hepatoprotective drugs than the currently available medications still exist. This strengthens the importance to discover novel therapeutic options, which can also tackle the socioeconomic burden of these diseases (Wang et al., 2021).

**FIGURE 1:**
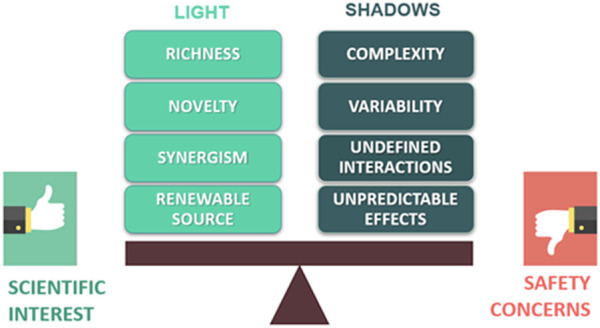
Scheme of the possible advantages and issues related to the use of plant-based natural products in drug discovery.

In this context, a renewed strategy is represented by plant-based natural products, which provide a wide range of new and unique bioactive molecules for drug discovery (Figure 1). These phytochemicals can be responsible for the healing properties of the extracts both by acting synergistically within the phytocomplex or as pure compounds. Moreover, herbal by-products and waste could be exploited as renewable sources of bioactive natural substances (Di Sotto et al., 2019).

However, “all that glitters is not gold”! Indeed, searching for new drug candidates from natural products is often limited by some issues that can also raise safety concerns. Particularly, the complexity and variability of phytochemical composition could lead to unpredictable and unexpected effects, along with possible interactions with concomitantly administered drugs (Figure 1). In this context, using phytochemically characterized and standardized herbal extracts can allow to partially control the undesired effects and achieve reproducible bioactivities (Thomford et al., 2018).

The complex picture described above is, at least in part, the cause of the contrasting evidence about the relationship between natural products and hepatic health. Indeed, while some preclinical and clinical studies underpin a potential interest in botanicals to manage liver diseases (Wang et al., 2020), others highlight safety concerns associated with their use (Ippoliti et al., 2021). In line with this evidence, the articles collected within the Research Topic “Natural Products and Hepatic Health: Light and Shadows” can contribute to the advancement of our knowledge about the impact of botanicals on hepatic health. Particularly, some articles tried to provide solid scientific bases to the traditional use of certain botanicals. Yang et al. have studied the antihyperlipidemic effect of the Traditional Chinese Medicine (TCM) formula Qing Gan San (QGS) in a rat model of metabolic associated fatty liver disease. QGS exhibited hepatoprotective effects and lowered lipid accumulation in the liver, likely through the inhibition of sterol regulatory element-binding protein-1. Similarly, the TCM formula Liuweiwuling was found endowed with *in vitro* and *in vivo* hepatoprotective properties, mediated by anti-apoptotic, anti-inflammatory, and antioxidant effects (Gao et al.); esculetin, luteolin, schisandrin A and schisandrin B were highlighted to be the major bioactive phytochemicals. The pharmacological power of TCM formulas in liver diseases has been also described by Fu et al.; notably, they highlighted that TCM can relieve liver diseases through the modulation of several pathways among which Nrf2, NF-κB, PI3K/Akt, and IL-6/STAT3, leading to antiinflammatory, antioxidant and antiviral effects.

Nowadays, several studies have shown that liver health is strictly connected to gut microbiota homeostasis (Minemura and Shimizu, 2015). In this context, Zhang et al. studied the changes in gut microbiota induced by the TCM Yinchen Wuling powder and showed that this formula was able to increase the abundance of eubacteria, and modulate inflammation and immune function, thus improving liver health. Similarly, Yu et al. underpinned the anti-cholestatic effects of the TCM formula Zhuyu Pill, mediated by changes in amino acid metabolism, steroid hormone biosynthesis, and bile secretion, along with an increase in fecal eubacteria. A specific focus on the hepatoprotection by chlorogenic acid, through the modulation of gut microbiota and glucagon-like peptide-1, has been addressed by Shi et al. in an *in vivo* NAFLD model.

Herbal by-products and waste have been also approached as a source of hepatoprotective agents. In this context, the hepatoprotective effects of different extracts from *Punica granatum* L. peels, known to be a rich source of polyphenols, have been evaluated by Ali et al. in a rat model of carbon tetrachloride–induced liver injury. The tested samples affected the inflammatory and oxidative response, and the liver enzyme levels; such effects were like those of the standard silymarin. Similarly, Feng et al. investigated the potential usefulness of a polysaccharide-based extract from the *Schisandra chinensis* (Turcz.) Baill. caulis in a rat model of HFD-induced NAFLD. Interestingly, the extract was found able to improve the liver enzyme levels and lipid parameters, through the regulation of metabolic pathways, and oxidative stress inhibition.

Mixtures of different herbal extracts as possible remedies to fight liver diseases have been approached too. Particularly, Frigerio et al. dealt with the anticholesterolemic activity of a commercial formulation based on a blend of artichoke, caigua, and fenugreek extracts. The product was found able to reduce total and free cholesterol, and to increase bile acid synthesis, acting similarly to red yeast rice, thus strengthening the interest in its nutraceutical role. Chen et al. also highlighted the ability of a fixed mixture of geniposide and chlorogenic acid to blunt the hepatic *de novo* lipogenesis, by inhibiting stearoyl-CoA desaturase-1, in an *in vivo* model of NAFLD.

At last, in line with the importance to address the possible safety concerns related to botanical use, Stati et al. critically evaluated recent cases of hepatotoxicity linked to *Curcuma longa* L. food supplements. According to previously published evidence (Vitalone et al., 2011; Frenzel and Teschke, 2016), the authors pointed out that other factors related to the subject susceptibility or to the product composition and bioavailability should be considered in the causality assessment as possible contributors of liver damage.

Altogether the results of the articles collected in the present Research Topic greatly improve our knowledge about the effects of herbal products on liver function and the mechanisms involved, and suggest possible future developments in nutraceutical and pharmacological fields. However, more outstanding research, as those here collected, and clinical trials are still needed to clarify the opportunities and pitfalls of natural products in liver diseases so putting light in the shadows.
